# Cytokine levels in pleural fluid as markers of acute rejection after lung transplantation*

**DOI:** 10.1590/S1806-37132014000400011

**Published:** 2014

**Authors:** Priscila Cilene León Bueno de Camargo, José Eduardo Afonso, Marcos Naoyuki Samano, Milena Marques Pagliarelli Acencio, Leila Antonangelo, Ricardo Henrique de Oliveira Braga Teixeira

**Affiliations:** Department of Pulmonology, Heart Institute, University of São Paulo School of Medicine Hospital das Clínicas , São Paulo, Brazil; Department of Pulmonology, Heart Institute, University of São Paulo School of Medicine Hospital das Clínicas , São Paulo, Brazil; Department of Thoracic Surgery, Heart Institute, University of São Paulo School of Medicine Hospital das Clínicas , São Paulo, Brazil; Laboratory for Pleural Studies, Heart Institute, University of São Paulo School of Medicine Hospital das Clínicas, São Paulo, Brazil; Department of Cytology, Central Laboratory, University of São Paulo School of Medicine Hospital das Clínicas , São Paulo, Brazil; Lung Transplant Group, Heart Institute, University of São Paulo School of Medicine Hospital das Clínicas, São Paulo, Brazil

**Keywords:** Lung transplantation, Pleural effusion, Cytokines, Graft rejection

## Abstract

Our objective was to determine the levels of lactate dehydrogenase, IL-6, IL-8, and VEGF, as well as the total and differential cell counts, in the pleural fluid of lung transplant recipients, correlating those levels with the occurrence and severity of rejection. We analyzed pleural fluid samples collected from 18 patients at various time points (up to postoperative day 4). The levels of IL-6, IL-8, and VEGF tended to elevate in parallel with increases in the severity of rejection. Our results suggest that these levels are markers of acute graft rejection in lung transplant recipients.

Lung transplantation (LT) is a therapeutic option for patients with advanced lung disease. Despite advances in the treatment of lung transplant recipients, acute graft rejection remains common, affecting up to 55% of all such patients in the first postoperative year. Rejection is the major risk factor for bronchiolitis obliterans syndrome and appears to be related to a humoral response with complement activation and production of donor-specific HLA antibodies. ^(^ 1 ^)^ Transbronchial biopsy (TBB) is the primary method for diagnosing acute cellular rejection. Several studies have shown a correlation between elevated serum cytokine levels and postoperative complications, as well as an association of serum cytokine levels with reperfusion edema, acute rejection, and bronchiolitis obliterans syndrome.^(^ 2 ^)^ The objective of the present study was to determine whether acute cellular rejection correlates with lactate dehydrogenase (LDH) levels, proinflammatory cytokine levels, and differential cell counts in the pleural fluid of lung transplant recipients. We hypothesized that elevated levels of proinflammatory cytokines in the pleural fluid of lung transplant recipients are early indicators of graft rejection. 

Between August of 2006 and March of 2008, 20 lung transplant recipients were evaluated for inclusion in the present study, 18 being included. Two patients were excluded from the analysis because they died within the first 6 weeks. The overall LDH levels, overall cytokine levels, and cell counts in pleural fluid were reported elsewhere, without any reference to rejection.^(^ 3 ^)^

Blood and pleural fluid samples were collected 6 h after surgery and daily until postoperative day 4. A TBB was performed at 2 and 6 weeks after LT in order to evaluate the severity of rejection, which was classified according to the intensity of perivascular mononuclear cell infiltration in the lung parenchyma, as follows: A0, no rejection; A1, minimal rejection; A2, mild rejection; A3, moderate rejection; and A4, severe rejection. For the final analysis, the highest degree of rejection was selected. 

No TBB samples underwent blind analysis. All TBB procedures were performed by a trained pathologist as routinely done at our facility. At least 5 tissue samples were analyzed in order to ensure that the lung parenchyma was adequately represented. 

In 3, 4, 8, and 3 of the 18 patients studied, the severity of rejection was classified as A0, A1, A2, and A3, respectively. 

Serum levels of IL-6 and IL-8 were undetectable in all samples. Serum VEGF levels were much lower than pleural fluid VEGF levels and were not significantly different among subgroups A0, A1, A2, and A3. 

In the present study, undetectable serum cytokine levels suggest that the inflammatory response in lung transplant recipients is primarily confined to the lungs and pleural cavity. Similar results have been reported elsewhere,^(^ 2 ^,^ 4 ^,^ 5 ^)^ acute elevation of serum cytokine levels having been found to be more evident in patients with reperfusion edema or acute rejection. 

The correlation between LDH levels and differential cell counts in pleural fluid is shown in Table 1, having been found to be higher in the subgroups of patients with graft rejection. 

**Table 1:** Relationship of the severity of acute rejection with the levels of lactate dehydrogenase, as well as with the total and differential cell counts, in pleural fluid samples collected at various time points.a

Variable	Severity of acute rejection			
	A0	A1	A2	A3
LDH, mg/dL				
6 h	1,855 ± 594	3,176 ± 530**	2,310 ± 964	4,134 ± 1,487*
24 h	1,250 ± 1,028	1,605 ± 1,285	1,779 ± 1,233	2,214 ± 958
48 h	1,181 ± 293	1,455 ± 642	966 ± 503	1,638 ± 1,201
72 h	375 ± 46	655 ± 462	576 ± 159	711 ± 203***
96 h	316 ± 33	586 ± 434	498 ± 214	621 ± 233
Total cells, cells/mL				
6 h	1,679 ± 1,612	3,812 ± 2,387	4,606 ± 3,156	10,710 ± 6,394***
24 h	595 ± 786	2,764 ± 1,250****	2,359 ± 645****	4,723 ± 2,514****^,^*****
48 h	645 ± 116	897 ± 626	649 ± 421	2,457 ± 2,180
72 h	246 ± 323	316 ± 168	542 ± 456	599 ± 680
96 h	167 ± 223	418 ± 288	435 ± 399	482 ± 120***
Neutrophils				
6 h	1,573 ± 1,485	3,580 ± 2,281	4,368 ± 3,074	9,884 ± 5,245******
24 h	537 ± 707	2,471 ± 942****	1,314 ± 1,255****	4,478 ± 2,553****
48 h	588 ± 147	812 ± 642	590 ± 450	2,266 ± 2,255
72 h	210 ± 274	266 ± 155	475 ± 431	374 ± 393
96 h	144 ± 192	258 ± 252	367 ± 339	235 ± 195
Lymphocytes				
6 h	90 ± 112	161 ± 56	156 ± 126	1,078 ± 1,455
24 h	52 ± 72	136 ± 140	71 ± 28	191 ± 74***
48 h	45 ± 32	65 ± 62	48 ± 50	145 ± 90***
72 h	36 ± 50	45 ± 24	36 ± 4	133 ± 166
96 h	23 ± 32	78 ± 61	41 ± 43	59 ± 62

A0 : no rejection

A1 : minimal rejection

A2 : mild rejection

A3 : moderate rejection

LDH : lactate dehydrogenase

a Values expressed as mean ± SD

* p < 0.05 (A3 > A0 and A2)

** p < 0.05 (A1 > A0)

*** p < 0.05 (A3 > A0)

**** p < 0.05 (A3, A2, and A1 > A0)

***** p < 0.05 (A3 > A1 and A2)

****** p < 0.05 (A3 > A0, A1, and A2).

We found elevated levels of IL-6 and IL-8 in the pleural fluid samples collected 6 h after LT, those levels having decreased by postoperative day 4. No such variation was observed in VEGF levels. Patients in whom graft rejection was more severe tended to have higher levels of those cytokines (Table 2). Patients in whom the severity of rejection was classified as A3 had higher levels of IL-6 and IL-8 at all time points when compared with those in whom graft rejection was less severe. In the samples collected 6 h after LT, VEGF levels were higher in the patients in whom the severity of rejection was classified as A3, A2, or A1 than in those in whom it was classified as A0. 

**Table 2:** Relationship of the severity of acute rejection with the levels of IL-6, IL-8, and VEGF in pleural fluid samples collected at various time points.a

Variable	Severity of acute rejection			
	A0	A1	A2	A3
IL-6, pg/mL				
6 h	14,717 (10,719-18,816)	27,368 (20,445-41,316)	38,521 (29,543-45,367)	49,854 (42,854-53,415)*
24 h	6,384 (2,304-10,464)	13,410 (10,973-17,608)	12,060 (8,886-17,824)	21,337 (17,779-48,322)**
48 h	7,642 (2,707-12,576)	11,402 (9,370-13,303)	12,211 (9,075-13,477)	15,010 (12,717-46,503)**
72 h	5,010 (2,227-7,793)	7,731 (5,301-9,687)	4,891 (3,748-6,303)	8,506 (4,871-8,605)
96 h	2,879 (2,196-3,562)	7,372 (6,292-8,461)***	4,838 (3,963-8,047)***	6,151 (4,593-7,709)***
IL-8, pg/mL				
6 h	1,318 (1,020-1,617)	1,706 (1,018-1,956)	1,696 (1,286-1,941)	2,216 (2,110-2,323)****
24 h	1,266 (996-1,536)	1,177 (767-1,608)	1,224 (799-1,706)	2,187 (2,119-2,254)****
48 h	1,091 (760-1,423)	1,455 (765-1,523)	1,037 (788-1,169)	2,036 (1,922-2,150)****
72 h	965 (579-1,351)	1,472 (377-1,633)	945 (682-1,163)	1,935 (1,775-2,096)****
96 h	981 (482-1,481)	464 (244-1,127)	690 (288-1,005)	1,859 (1,775-1,943)****
VEGF, pg/mL				
6 h	72 (34-110)	343 (290-508)***	297 (145-504)***	566 (153-879)***
24 h	123 (18-228)	188 (115-284)	279 (77-445)	382 (107-745)
48 h	121 (20-221)	123 (95-172)	283 (84-435)	123 (54-406)
72 h	142 (17-267)	143 (112-214)	294 (116-382)	123 (39-406)
96 h	144 (8-280)	134 (115-336)	280 (191-425)	280 (81-445)

A0 : no rejection

A1 : minimal rejection

A2 : mild rejection

A3 : moderate rejection

a Values expressed as median (interquartile range)

* p < 0.05 (A3 > A0 and A1)

** p < 0.05 (A3 > A0)

*** p < 0.05 (A3, A2, and A1 > A0)

**** p < 0.05 (A3 > A2, A1, and A0).

Inflammation of the pleural space is due to the surgical trauma and the presence of a chest tube and can explain the high levels of cytokines. In our study, it is of note that, although inflammatory marker levels progressively decreased over time, proinflammatory cytokine levels remained elevated during the first 4 days, and irritation caused by the chest tube left in place for up to 96 h can explain that, although it does not explain the differences among the subgroups of patients. We speculate that the cytokine levels found in the patients classified as A0 represent the increase in cytokine levels that occurs as a result of the surgical trauma and the use of chest tubes. This level of inflammation decreases over time, as evidenced by a reduction in pleural fluid levels of IL-6 and IL-8. The cytokine levels found in the patients in whom the severity of rejection was classified as A3 were much higher than were those found in the remaining subgroups of patients, and this might be an early indicator of rejection. 

Pleural fluid levels of IL-6 correlated positively with LDH levels (r = 0.49; p = 0.030) and the neutrophil count (r = 0.90; p = 0.036), and VEGF levels were strongly correlated with the neutrophil count (r = 0.91; p = 0.030) and the total leukocyte count (r = 0.88; p = 0.048). In contrast, a strong negative correlation was found between IL-8 levels and the lymphocyte count (r = −0.97; p = 0.007). In addition, a strong positive correlation was found between IL-6 levels and IL-8 levels (r = 0.70; p < 0.001), as well as between IL-6 levels and VEGF levels (r = 0.71; p < 0.001). Furthermore, there was a slight correlation between IL-8 levels and VEGF levels (r = 0.49; p = 0.027). In the present study, there was no correlation between the severity of acute rejection and the severity of primary graft dysfunction within the first 72 h after LT. 

In lung transplant recipients, inflammation of the lung parenchyma leads to increased interstitial edema with increased cytokine levels. Because lymphatic vessels are cut, the amount of interstitial fluid leaving the lungs and going through the visceral pleura increases. This can lead to high cytokine levels, which have been observed in patients with graft rejection. 

One limitation of the present study was the small number of patients in each subgroup. However, it is clear that inflammatory cytokine levels were highest in the patients in whom the severity of rejection was classified as A3. The lack of a significant difference between subgroups A1 and A2 regarding cytokine levels is possibly due to the small sample size. In addition, the fact that pleural fluid collection and TBB occurred at different time points possibly influenced the results. The data presented here are old, and a larger study, based on these data, is currently under way. 

The present study demonstrated that elevated levels of proinflammatory cytokines in the pleural fluid of lung transplant recipients can be markers of acute graft rejection (particularly of moderate to severe rejection). In this context, determination of cytokine levels in pleural fluid can be useful in identifying patients who will develop graft rejection. Therefore, treatment can be initiated sooner, thus reducing lung parenchymal injury. 
