# Supplementary material for: Cytokine levels in pleural fluid as markers of acute rejection after lung transplantation
Source: J Bras Pneumol. 2014 Jul-Aug;40(4):425–8. doi: 10.1590/S1806-37132014000400011 (PMC4201174; doi:10.1590/S1806-37132014000400011)
Supplement: Translation

# Translation: Citocinas no líquido pleural após transplante pulmonar como marcadores de rejeição aguda*

**Authors:** Priscila Cilene León Bueno de Camargo, José Eduardo Afonso, Marcos Naoyuki Samano, Milena Marques Pagliarelli Acencio, Leila Antonangelo, Ricardo Henrique de Oliveira Braga Teixeira

**Affiliations:** Divisão de Pneumologia, Instituto do Coração, Hospital das Clínicas, Faculdade de Medicina da Universidade de São Paulo, São Paulo (SP) Brasil; Divisão de Pneumologia, Instituto do Coração, Hospital das Clínicas, Faculdade de Medicina da Universidade de São Paulo, São Paulo (SP) Brasil; Divisão de Cirurgia Torácica, Instituto do Coração, Hospital das Clínicas, Faculdade de Medicina da Universidade de São Paulo, São Paulo (SP) Brasil; Laboratório de Pleura, Instituto do Coração, Hospital das Clínicas, Faculdade de Medicina da Universidade de São Paulo, São Paulo (SP) Brasil; Serviço de Citologia, Divisão de Laboratório Central, Hospital das Clínicas, Faculdade de Medicina da Universidade de São Paulo, São Paulo (SP) Brasil; Grupo de Transplante de Pulmão, Instituto do Coração, Hospital das Clínicas, Faculdade de Medicina da Universidade de São Paulo, São Paulo (SP) Brasil

**Keywords:** Transplante de pulmão, Derrame pleural, Citocinas, Rejeição de enxerto

## Abstract

Nosso objetivo foi determinar os níveis de desidrogenase lática, IL-6. IL-8 e VEGF, assim como a contagem total e diferencial de células no líquido pleural de transplantados de pulmão, correlacionando esses níveis com a ocorrência e a gravidade de rejeição após o procedimento. Foram analisadas amostras de líquido pleural coletadas de 18 pacientes em diferentes momentos (até o quarto dia pós-operatório). Os níveis de IL-6, IL-8 e VEGF apresentaram uma tendência de aumento paralelamente à gravidade de rejeição. Nossos resultados sugerem que esses níveis são indicadores de rejeição aguda do enxerto em transplantados de pulmão.

O transplante pulmonar (TP) é uma escolha terapêutica para pacientes com pneumopatias avançadas. Apesar do avanço no tratamento dos transplantados pulmonares, a rejeição aguda do enxerto ainda permanece comum, afetando até 55% desses pacientes no primeiro ano pós-operatório. A rejeição é o principal fator de risco para o desenvolvimento da síndrome de bronquiolite obliterante e parece estar relacionada com o desenvolvimento de resposta humoral com ativação de complemento e produção de anticorpos HLA contra o doador.^(^ 1 ^)^ A biópsia transbrônquica (BTB) é o principal método para o diagnóstico de rejeição celular aguda. Vários estudos têm mostrado a correlação entre níveis séricos elevados de citocinas e complicações no pós-operatório, além da associação desses níveis com edema de reperfusão, rejeição aguda e síndrome de bronquiolite obliterante.^(^ 2 ^)^ O objetivo do presente estudo foi correlacionar os níveis de desidrogenase lática (DHL) e de citocinas pró-inflamatórias, assim como a contagem diferencial de células, no líquido pleural com a rejeição celular aguda em pacientes submetidos a TP. Nossa hipótese era de que níveis elevados de citocinas pró-inflamatórias no líquido pleural seriam indicadores precoces de rejeição do enxerto no TP.

Entre agosto de 2006 e março de 2008, 20 pacientes submetidos a TP foram avaliados no estudo, sendo 18 incluídos. Dois pacientes foram excluídos da análise porque morreram nas primeiras seis semanas. Os valores globais de DHL e de citocinas e a contagem de células no líquido pleural, sem qualquer referência à rejeição, foram relatados em um artigo anterior.^(^ 3 ^)^

Amostras de sangue e de líquido pleural foram recolhidas para avaliação 6 h após a cirurgia e diariamente até o 4º dia pós-operatório. Realizamos BTB na segunda e sexta semanas após o TP para a avaliação de rejeição, utilizando um critério de classificação (A0 = sem rejeição; A1 = rejeição mínima; A2 = rejeição leve; A3 = rejeição moderada; e A4 = rejeição grave) de acordo com a intensidade de infiltrado mononuclear perivascular no parênquima pulmonar. Para a análise final, o paciente foi classificado de acordo com o grau mais grave de rejeição nas duas biópsias. 

Não foi realizada uma análise cega das biópsias, as quais seguiram a rotina assistencial do serviço por um patologista treinado, sendo analisadas pelo menos 5 amostras de tecido para garantir a representatividade do parênquima pulmonar.

Dos 18 pacientes do estudo, 3, 4, 8 e 3, respectivamente, foram classificados nas categorias A0, A1, A2 e A3.

Os níveis séricos de IL-6 e IL-8 foram indetectáveis em todas as amostras. Os níveis séricos de VEGF foram muito menores do que os níveis de VEGF no líquido pleural e não diferiram significativamente nos pacientes com diferentes graus de rejeição.

No presente estudo, os níveis séricos indetectáveis de citocinas sugerem que a resposta inflamatória no TP pode estar confinada primariamente no pulmão e na cavidade pleural. Resultados semelhantes foram relatados em outros estudos,^(^ 2 ^,^ 4 ^,^ 5 ^)^ observando-se que a elevação aguda dessas citocinas foi mais evidente nos pacientes que tiveram edema de reperfusão ou rejeição aguda.

A correlação entre os níveis de DHL e a contagem diferencial de células no líquido pleural está descrita na Tabela 1, apresentando valores maiores nos grupos de pacientes com rejeição.

**Tabela 1:** Relação dos níveis de desidrogenase lática e das contagens totais e diferenciais de células em amostras de líquido pleural coletadas em diferentes momentos com o grau de rejeição aguda.a

Variáveis	Grau de rejeição aguda			
	A0	A1	A2	A3
DHL, mg/dL				
6 h	1.855 ± 594	3.176 ± 530**	2.310 ± 964	4.134 ± 1.487*
24 h	1.250 ± 1.028	1.605 ± 1.285	1.779 ± 1.233	2.214 ± 958
48 h	1.181 ± 293	1.455 ± 642	966 ± 503	1.638 ± 1.201
72 h	375 ± 46	655 ± 462	576 ± 159	711 ± 203***
96 h	316 ± 33	586 ± 434	498 ± 214	621 ± 233
Células totais, cels/mL				
6 h	1.679 ± 1.612	3.812 ± 2.387	4.606 ± 3.156	10.710 ± 6.394***
24 h	595 ± 786	2.764 ± 1.250****	2.359 ± 645****	4.723 ± 2.514****^.^*****
48 h	645 ± 116	897 ± 626	649 ± 421	2.457 ± 2.180
72 h	246 ± 323	316 ± 168	542 ± 456	599 ± 680
96 h	167 ± 223	418 ± 288	435 ± 399	482 ± 120***
Neutrófilos				
6 h	1.573 ± 1.485	3.580 ± 2.281	4.368 ± 3.074	9.884 ± 5.245******
24 h	537 ± 707	2.471 ± 942****	1.314 ± 1.255****	4.478 ± 2.553****
48 h	588 ± 147	812 ± 642	590 ± 450	2.266 ± 2.255
72 h	210 ± 274	266 ± 155	475 ± 431	374 ± 393
96 h	144 ± 192	258 ± 252	367 ± 339	235 ± 195
Linfócitos				
6 h	90 ± 112	161 ± 56	156 ± 126	1.078 ± 1.455
24 h	52 ± 72	136 ± 140	71 ± 28	191 ± 74***
48 h	45 ± 32	65 ± 62	48 ± 50	145 ± 90***
72 h	36 ± 50	45 ± 24	36 ± 4	133 ± 166
96 h	23 ± 32	78 ± 61	41 ± 43	59 ± 62

A0 : sem rejeição

A1 : rejeição mínima

A2 : rejeição leve

A3 : rejeição moderada

DLH : desidrogenase lática

a Valores expressos em média ± dp

* p < 0.05 (A3 > A0 e A2)

** p < 0.05 (A1 > A0)

*** p < 0.05 (A3 > A0)

**** p < 0.05 (A3, A2 e A1 > A0)

***** p < 0.05 (A3 > A1 e A2)

****** p < 0.05 (A3 > A0, A1 e A2).

Encontramos níveis elevados de IL-6 e IL-8 no líquido pleural amostrado 6 h após o TP, com subsequente redução até o 4º dia pós-operatório. Tal variação não ocorreu com os níveis de VEGF. Pacientes com níveis mais elevados de rejeição tenderam a ter níveis mais elevados dessas citocinas (Tabela 2). Pacientes classificados como A3 tinham níveis mais elevados de IL-6 e IL-8 em todos os momentos quando comparados com aqueles com graus mais baixos de rejeição. Os níveis de VEGF foram maiores para os pacientes dos grupos A3, A2 e A1 quando comparados com aqueles do grupo A0 na amostra retirada após 6 h do TP.

**Tabela 2:** Relação dos níveis de IL-6, IL-8 e VEGF em amostras de líquido pleural coletadas em diferentes momentos com o grau de rejeição aguda.a

Variáveis	Grau de rejeição aguda			
	A0	A1	A2	A3
IL-6, pg/mL				
6 h	14.717 (10.719-18.816)	27.368 (20.445-41.316)	38.521 (29.543-45.367)	49.854 (42.854-53.415)*
24 h	6.384 (2.304-10.464)	13.410 (10.973-17.608)	12.060 (8.886-17.824)	21.337 (17.779-48.322)**
48 h	7.642 (2.707-12.576)	11.402 (9.370-13.303)	12.211 (9.075-13.477)	15.010 (12.717-46.503)**
72 h	5.010 (2.227-7.793)	7.731 (5.301-9.687)	4.891 (3.748-6.303)	8.506 (4.871-8.605)
96 h	2.879 (2.196-3.562)	7.372 (6.292-8.461)***	4.838 (3.963-8.047)***	6.151 (4.593-7.709)***
IL-8, pg/mL				
6 h	1.318 (1.020-1.617)	1.706 (1.018-1.956)	1.696 (1.286-1.941)	2.216 (2.110-2.323)****
24 h	1.266 (996-1.536)	1.177 (767-1.608)	1.224 (799-1.706)	2.187 (2.119-2.254)****
48 h	1.091 (760-1.423)	1.455 (765-1.523)	1.037 (788-1.169)	2.036 (1.922-2.150)****
72 h	965 (579-1.351)	1.472 (377-1.633)	945 (682-1.163)	1.935 (1.775-2.096)****
96 h	981 (482-1.481)	464 (244-1.127)	690 (288-1.005)	1.859 (1.775-1.943)****
VEGF, pg/mL				
6 h	72 (34-110)	343 (290-508)***	297 (145-504)***	566 (153-879)***
24 h	123 (18-228)	188 (115-284)	279 (77-445)	382 (107-745)
48 h	121 (20-221)	123 (95-172)	283 (84-435)	123 (54-406)
72 h	142 (17-267)	143 (112-214)	294 (116-382)	123 (39-406)
96 h	144 (8-280)	134 (115-336)	280 (191-425)	280 (81-445)

A0 : sem rejeição

A1 : rejeição mínima

A2 : rejeição leve

A3 : rejeição moderada

a Valores expressos em mediana (intervalo interquartílico)

* p< 0.05 (A3 > A0 e A1)

** p < 0.05 (A3 > A0)

*** p < 0.05 (A3, A2 e A1 > A0)

**** p < 0.05 (A3 > A2, A1 e A0).

Devido ao trauma cirúrgico e a presença de um dreno, há inflamação do espaço pleural, o que poderia explicar os níveis elevados de citocinas. Em nosso estudo, é interessante notar que, apesar da redução progressiva dos marcadores inflamatórios com o tempo, os níveis de citocinas pró-inflamatórias permaneceram elevados durante os quatro primeiros dias, e a irritação causada pelo dreno deixado no local por até 96 h é uma possível explicação para esse fato, o qual, no entanto, não explica as diferenças observadas nos subgrupos. Especulamos que os níveis de citocinas presentes nos pacientes considerados A0 representam o aumento de citocinas resultante do trauma cirúrgico e do uso de drenos. Esse nível de inflamação diminui com o tempo, como foi evidenciado pela redução nos níveis de IL-6 e IL-8 no líquido pleural. Os níveis de citocinas nos pacientes do subgrupo A3 foram muito mais elevados do que nos outros subgrupos de rejeição, o que pode ser um indicador precoce de rejeição. 

Foram encontradas correlações positivas entre os níveis de IL-6 com os níveis de DHL no líquido pleural (r = 0,49; p = 0,030) e com a contagem de neutrófilos (r = 0,90; p = 0,036), enquanto os níveis de VEGF correlacionaram-se fortemente com a contagem de neutrófilos (r = 0,91; p = 0,030) e de leucócitos totais (r = 0,88; p = 0,048). Por outro lado, foi encontrada uma forte correlação negativa entre os níveis de IL-8 no líquido pleural e a contagem de linfócitos (r = −0,97; p = 0,007). Além disso, uma forte correlação positiva foi encontrada entre os níveis de IL-6 no líquido pleural com aqueles de IL-8 (r = 0,70; p < 0,001) e de VEGF (r = 0,71; p < 0,001), assim como houve uma ligeira correlação entre os níveis de IL-8 e de VEGF (r = 0,49; p = 0,027). No presente estudo, não houve correlação entre o grau de rejeição aguda e o grau de disfunção do enxerto primário nas primeiras 72 h após o TP.

Nos pacientes submetidos a TP, a inflamação do parênquima pulmonar conduz a um aumento do edema intersticial, com níveis elevados de citocinas. Uma vez que os vasos linfáticos são cortados, mais fluido intersticial sai dos pulmões, atravessando a pleura visceral, podendo levar a níveis elevados de citocinas, que foram observados nos pacientes com rejeição.

Uma limitação do estudo foi o pequeno número de pacientes em cada subgrupo. No entanto, é evidente que os níveis de citocinas inflamatórias foram mais elevados nos pacientes com grau de rejeição A3 em comparação com aqueles de outros subgrupos. Destacamos também que o pequeno tamanho da amostra pode ser responsável pela falta de significância estatística observada nos níveis de citocinas entre os pacientes dos subgrupos A1 e A2. Além disso, os resultados podem ter sido influenciados pelo fato de que a coleta do líquido pleural e a BTB ocorreram em momentos distintos. Esses dados são antigos, e um estudo maior baseado neles está em andamento.

Demonstramos que níveis elevados de citocinas pró-inflamatórias no líquido pleural de pacientes submetidos a TP podem ser marcadores de rejeição aguda do enxerto, em particular, de grau moderado a severo. Nesse contexto, é possível que a determinação de citocinas no líquido pleural seja útil na identificação de pacientes que irão desenvolver rejeição. Dessa forma, o tratamento poderia ser iniciado mais cedo, levando a redução da lesão no parênquima pulmonar.
